# The Cancer Rehabilitation Medicine Metrics Consortium: A Path to Enhanced, Multi-Site Outcome Assessment to Enhance Care and Demonstrate Value

**DOI:** 10.3389/fonc.2020.625700

**Published:** 2021-02-03

**Authors:** Sean R. Smith, Mary Vargo, David S. Zucker, Maryanne Henderson, Samman Shahpar, Eric M. Wisotzky, Christian Custodio, Jeffrey Basford, Gina Jay, Lynn Gerber, Andrea Cheville

**Affiliations:** ^1^ Department of Physical Medicine and Rehabilitation, Michigan Medicine, Ann Arbor, MI, United States; ^2^ Case Western Reserve University, Cleveland, OH, United States; ^3^ Cancer Rehabilitation Medicine Services, Swedish Cancer Institute, Swedish Health Services, Seattle, WA, United States; ^4^ Department of Physical Medicine and Rehabilitation, University of Pittsburgh Medical Center, Pittsburgh, PA, United States; ^5^ Shirley Ryan AbilityLab, Chicago, IL, United States; ^6^ MedStar National Rehabilitation Hospital, Washington, DC, United States; ^7^ Department of Neurology, Memorial Sloan Kettering Cancer Center, New York, NY, United States; ^8^ Department of Physical Medicine and Rehabilitation, Mayo Clinic, Rochester, MN, United States; ^9^ Center for Study of Chronic Illness and Disability, George Mason University, Fairfax, VA, United States

**Keywords:** cancer rehabilitation, patient reported outcome measures, outcome measurement, cancer function, functional measurement, cancer survivorship

## Abstract

**Purpose:**

A primary objective stated at the Cancer Rehabilitation Symposium at the National Institutes of Health was to improve outcome measurement. The purpose of this project was for the Cancer Rehabilitation Medicine Metrics Consortium (CRMMC) to develop an assessment tool to evaluate function in cancer patients via a data-driven and methodologically sound process. There is no agreed-upon measure of physical and cognitive function for cancer patients, making it difficult to demonstrate the value of rehabilitation interventions. Cancer patients are a particularly challenging population, with many tumor- and treatment-related variables impacting function.

**Methods:**

Investigators from nine different cancer rehabilitation programs participated in a modified-Delphi process to delineate necessary aspects of an ideal patient assessment tool, including instrument type, domains evaluated, applicability across a range of patient traits, clinical feasibility, and item response characteristics. This involved numerous meetings, data review, and analysis of items involved in patient assessment.

**Results:**

The CRMMC developed a 21-item patient-reported outcome measure based on item response theory. The process by which the short form was developed was documented and provides a framework for other clinicians to follow.

**Conclusion:**

This document provides a framework for rehabilitation providers to follow when developing an assessment tool. This process is described in a stepwise fashion for reproducibility even in different, non-cancer populations.

## Introduction

There are over 15 million cancer survivors, many of whom are at risk for cancer-related disablement (1). Identifying and addressing their rehabilitation needs is essential and, unfortunately, a relatively under-utilized aspect of care (2, 3).

The field of cancer rehabilitation has grown in parallel with the rapid expansion of the cancer survivor population, however, its expansion has been characterized by programs in large tertiary and quaternary centers that may provide disparate services (4). This has left a knowledge gap regarding best practices and the individualization of care. The lack of agreement about use of outcome measures limits efforts to improve care as well as demonstrate the value of cancer rehabilitation to patients, oncologists, and payers alike (5, 6).

The need to capture clinical outcomes among cancer rehabilitation providers parallels the drive for transparent outcome reporting, influenced by patient needs and desires, to permit value-based health care purchasing and patient centered outcomes. Growing support among policy makers and other stakeholders for explicit linkages between reimbursement and clinical outcomes renders the methods of patient assessment an increasingly high stakes decision. Appropriate outcomes will help showcase the value of care while enabling providers to deliver more individualized treatment and measure its effectiveness.

There is a vast and steadily increasing array of outcome measures attempting to capture meaningful improvements afforded by cancer care. The potential consequences of poor selections, however, are not trivial; failure to demonstrate measurable benefit may cripple or prevent delivery of even the most beneficial care. Therefore, ensuring that the assessments used in cancer rehabilitation practice are capable of capturing and demonstrating value should be an uncontested priority. In short, outcome-related decision-making is not simple, but will become progressively more important to rehabilitation service providers.

To that end, the Cancer Rehabilitation Symposium at the National Institutes of Health designated improving patient-reported outcome measures as one of four essential components in the strategic plan to enhance cancer rehabilitation care (7). The Cancer Rehabilitation Medicine Metrics Consortium (CRMMC) (see **Table 1** for institutional membership) was formed following the symposium by a group of rehabilitation medicine physicians (physiatrists) specialized in cancer rehabilitation with the goal of selecting valid, reliable function driven outcome measures that would be patient-centered and capture domains relevant to oncology rehabilitation practice. The CRMMC’s long-term goal is to produce a multi-site data collection strategy that will enhance members’ ability to monitor care at the patient-level, while producing an aggregate data set that can be interrogated using artificial intelligence and other analytic tools to forecast population- and patient-level outcome trajectories. This manuscript outlines the sequence and specifics of decision-making regarding outcome selection followed by the CRMMC towards this goal. Methods are described by which rehabilitation service providers with a shared desire to simultaneously individualize treatment and describe population-level trends may select outcomes that improve clinical decision making and demonstrate value.

**Table 1:** Participating Institutions.

Case Western Reserve University/MetroHealth (Cleveland, OH)*	
George Mason University (Fairfax, VA)	
The Mayo Clinic (Rochester, MN)*	
MedStar National Rehabilitation Hospital (Washington, DC)	
Memorial Sloan Kettering Cancer Center (New York, NY)	
The Shirley Ryan Ability Lab (Chicago, IL)*	
Swedish Cancer Institute (Seattle, WA)*	
University of Michigan (Ann Arbor, MI)*^†^	
University of Pittsburgh Medical Center (Pittsburgh, PA)*	

*Data collection site; ^†^Data coordinating site.

## Methods

### CRMMC Formation, Structure, and Meeting Schedule

Investigators from nine different institutions formed the CRMMC with the goal of identifying a measurement tool to capture changes in domains relevant to cancer rehabilitation services (**Table 1**). The target population was patients receiving care for cancer-related functional morbidity or adverse symptoms in cancer rehabilitation outpatient clinics.

Regular telephone conferences were implemented to discuss strategies to develop the tool, write a protocol that would permit multi-site data collection, and eventually collect and analyze data from participant sites. More detail about each meeting’s topic is listed in **Appendix 1**.

The CRMMC used serial modified Delphi processes in which consensus among clinical experts was sought through a series of questions to devise the best recommendation for a given problem (outcome measure, clinical guideline, study design, etc.). This process was chosen based on precedent among prior groups in selecting tools to measure function and other outcomes (8–10). For the CRMMC, group decision-making progressed over a series of multiple telephone conferences to forge consensus regarding choice of measurement system, domains, items, and implementation strategy. Members completed independent analyses and reviews prior to each session. Between telephone conferences, additional communication was also carried out amongst CRMMC members *via* email.

### Conceptual Background

Physiatric practice is unique among medical specialties in that it addresses compromised whole-person function—that is, the use of one’s body and associated activity and participation—in real-time. Interventions require skills that target specific components of a survivor’s unique disability presentation with the dual outcome goals of reduced suffering and enhanced functioning. Barriers to optimal functioning are in part mediated by symptoms and/or impairments, frequently in clusters (11, 12) that disrupt interactions with social environments and present as disablement. The disability continuum is broad, ranging from profound activity limitations to high-level, minimally problematic functioning. Its evolution can be framed as a cascading, dynamic series of events beginning with disruption of normal physiology and anatomy, conceptualized as symptoms and/or impairments that undermine activity which, in turn, limit social participation. Moreover, complex, bidirectional interactions across this continuum produce dynamic, often shifting, clinical presentations (13).

Successful intervention hinges on accurately identifying and diagnosing key components of disablement at multiple points throughout each patient’s cancer care trajectory. This is critical, not only to improve clinical decision-making and to match intervention to presentation, but also to demonstrate the value of an intervention. The CRMMC’s approach to developing the assessment tool is grounded in this broad conceptual framework, derived from the International Classification of Function (ICF) as well as from physiatric training and practice principles. The CRMMC believes that this framework is appropriately broad to guide development of an instrument that is sufficiently robust to precisely map disablement domains and trait ranges to observed function, meet the high stakes nature of this enterprise, and to address unmet rehabilitation needs of cancer patients. This conceptual framework informs the selection of instruments and items, identifying subdomains, and defining trait ranges that follow.

### Delineation of Measurement Priorities and Specifics

The CRMMC set out to create a measure that would satisfy multiple clinical and research needs while minimizing response burden in a patient population already inundated with questionnaires. Foremost, the group wanted to construct a measure that would accurately report a patient’s function as it relates to their cancer-related impairments. Many assessment tools that purport to evaluate function ask about symptoms such as nausea that are at best indirect contributing factors to a change in patients’ physical and cognitive abilities and are not modifiable with rehabilitation interventions (e.g. Functional Assessment of Cancer Therapy - General) (14).

From a practical standpoint, the tool had to be feasible to administer in a busy clinical setting without significantly disrupting provider workflow. Additionally, the instrument must be able to be administered in various practice settings; an objective assessment requiring specialized equipment, for example, may not be feasible at performance sites with resource constraints.

The planned setting to administer this assessment would be in outpatient adult cancer rehabilitation clinics, and ideally eventually also in non-physician clinics and oncology clinics. Potentially, it would also be useful in a research setting to provide function-specific data. Finally, as this measure would only evaluate traits potentially amenable to rehabilitation intervention, it may be useful for triaging referrals to rehabilitation. For example, someone who reports poor physical function on this assessment may be appropriate for evaluation by a physiatrist. This would, however, require a study framework distinct from the validation process, and was beyond the scope of the initial development of the tool.

### Selection of Measurement Approach

Measurement is a requirement for identifying functional abnormalities and must be included in assessing the outcomes of rehabilitation interventions. The overall approach required integrating multi-domain inquiry (e.g. mobility, self-care, and social integration/participation) and site adoption. The factors influencing selection of measurements took into consideration the following important practical and theoretical considerations: a. reliability, b. validity, c. ease of use, d. applicability to the outpatient population, e. sensitivity to change, and f. clinical relevance in that it addressed function and social integration. Several options for the approach were considered, including:

#### Performance Based Measures

These measures include, grip strength, a timed walk test, and the Timed Up and Go test. They offer objective measures and may correlate with patient reported outcomes (PROs). A timed walk test or grip strength were strongly considered, but were not ultimately selected due to the challenges of obtaining them consistently in busy clinics at diverse institutions with varying resource availabilities.

#### Clinician Reported Outcomes (CROs)

CROs have constraints similar to performance measures: requirements for clinic-based assessment, human resource investment, and training. Additionally, some CROs have certification requirements, e.g., the Functional Independence Measure. More problematic than these limitations is an absence of candidate CROs for assessing the functional status of patients with cancer described in the peer-reviewed literature. The Karnofsky Performance Status (KPS) (15) and Eastern Cooperative Oncology Group (ECOG) (16) scales, routinely used in medical oncology, assess “performance status,” a construct correlated to but distinct from physical function. These two scales do not provide information about social participation and lack sufficient sensitivity to be used in repeated measure designed studies.

#### Activity Monitoring

Activity assessments using pedometers, actigraphs, and other motion capture sensors have become feasible, yet lack data ingestion, integration, and presentation standards, which limits their use in routine clinical practice. They also would require an indeterminate amount of time being worn by patients, making their utility questionable even if there was a feasible way to extract data for routine clinical use.

#### Patient Reported Outcomes

This option was thought best to reliably measure specific areas of movement incorporating functional activity important to patients, such as dressing, daily activity, and mobility. Further, it offers patients an opportunity to report ease or difficulty with participation and social activity. The psychometric properties of a select, limited number of questions that would make this approach feasible in busy clinic settings, similar to a CRO such as KPS or ECOG.

PROs have gained widespread acceptance with recognition of the importance of patient-centered care and knowledge that their use can improve care delivery, outcomes, and survival (17, 18). Additionally, many providers have turned to PROs as a cost-sensitive and scalable means to capture outcomes. There is compelling rationale for this focus as PROs have been shown to have measurement properties comparable or superior to CROs and performance measures (19), and to lend themselves to remote collection modes, e.g., web-based and SMS text. Additionally, PROs have created unprecedented opportunities for standardized collection and sharing of data across health care organizations (20). For these reasons, the CRMMC opted to use a PRO approach for patient assessment.

The CRMMC considered what properties the PRO tool should have to increase the chance of being used and provide useful, reliable data. Consensus was to limit the number of questions to roughly 10–20 items; ensure utility in measuring treatment outcomes for clinicians and other stakeholders, including payers; and be psychometrically vetted in the target population.

### Item Response Theory-Based or Legacy Instrument

The availability of item response theory (IRT)-modeled items banks may offer a potential solution to the challenge of developing an assessment tool that meets the CRMMC’s requirements. IRT-modeled item banks were chosen over legacy instruments given advantages of item-level information statistics, ratio data, coverage of entire trait ranges, and flexibility in administration, e.g., computerized adaptive test, standardized short forms (SFs), and customized SFs.

Several IRT-modeled banks are currently available that assess function. These include the Activity Measure for Post-Acute Care (AM-PAC) system (21) and the Patient-Reported Outcome Measurement Information System (PROMIS) (22, 23). While the AM-PAC banks have been shown to be responsive among cancer populations (24), they do not include domains considered integral to clinical decision making and outcome assessment by outpatient cancer rehabilitation providers. In contrast, the PROMIS banks are increasingly used in diverse primary and specialty setting.

Further, PROMIS items banks were selected as the source of items for the CRMMC measure, in part because their items are highly applicable to the outpatient setting which is the main focus in this study, and the item banks are freely available in the public domain. The CRMMC also found that PROMIS items have a high density in the relevant trait ranges, align well with clinical issues among cancer survivors, and span the trait range of patients’ functional capacity, including that of higher-performing patients desiring to return to complex vocational settings. Additionally, the PROMIS may be more familiar to oncology clinicians than other options that are typically administered in a rehabilitation setting. Another benefit was the potential to contribute to the IRT field by replicating the initial calibrations among a cancer rehabilitation cohort, and providing an additional use case highlighting the versatility of the PROMIS banks.

PROMIS includes item banks spanning diverse domains, such as pain, fatigue, and social role functioning, each of which is comprised of items calibrated on a unidimensional trait continuum (25). The banks offer salient advantages including a dramatic lessening of ceiling and floor effects. Additionally, and most relevant to the CRMMC’s goal, IRT-modeled banks allow for the creation of SFs that can be tailored to assess the unique subdomains and trait ranges of specific clinical populations. Irrespective of the items chosen, these SFs yield scores comparable to other measures derived from the bank. Specifically, computerized adaptive testing (CAT), as well as standardized and custom SFs that include entirely different items from the same bank produce comparable scores on a common scale. The development of PROMIS banks thus represents a major conceptual shift in outcome measurement.

Short forms from multiple PROMIS domains can be combined to generate questionnaires that match the specific needs of a given clinical practice or population. Rigorous assessment of the PROMIS tools’ psychometric performance is ongoing and, thus far, they perform equally well, if not better than legacy measures with a correspondingly reduction in time and respondent burden (26–28). Additionally, the PROMIS banks offer the advantage, shared by all IRT-modeled banks, of simultaneously estimating measurement error and trait level. Awareness of measurement error is particularly beneficial in the assessment of rehabilitation populations who may have cognitive dysfunction and therefore provide inconsistent responses. Unlike legacy measures, IRT-derived tools enable clinicians to reliably identify when patient-reported information is unreliable and may indicate a need for objective testing or other evaluation.

There is a PROMIS cancer-specific physical function bank, however it consists of over 40 items, has limited subdomain coverage, and yields scores similar to the more robust and much larger general PROMIS bank. While it may be appropriate for some research applications, it is neither practical nor relevant to the needs of clinical care (29). Some of the measures selected for testing based on this process are included in the PROMIS – Ca Bank v1.1 – Physical Function form, so relevant items in the larger, existing PROMIS form will still be captured in this new tool.

Additionally, PROMIS has cancer-specific short forms addressing other symptoms including fatigue and mental health. These questionnaires are also very long (PROMIS – Ca Item Bank v1.0 – Fatigue alone has over 40 items) and relevant items from each short form were selected in this process to be studied in cancer rehabilitation patients. Items dealing solely with mental health (e.g. how frightened a patient feels) were not selected, as these would be more appropriate for a mental health provider to be evaluating rather than a rehabilitation provider.

### Selection of Domains

Through the modified Delphi process, the CRMMC decided to focus on four PROMIS domains: physical function, upper extremity function, fatigue, and ability to participate in social roles and activities. The CRMMC reached consensus that these four domains were most applicable to the functional morbidity experienced by cancer patients during and after treatment. Patients have routinely rated maintaining function as an important goal of care, and fatigue as one of the most bothersome symptoms (30–33). In addition, these issues are routinely managed in outpatient rehabilitation clinic settings. The CRMMC discussed including a composite quality of life assessment, but agreed that it would be unlikely to detect change in domains of interest, further compounded by potential signal loss in aggregate scores. Additionally, issues related to gastrointestinal function, sexual health, and other symptoms not necessarily addressed by rehabilitation interventions were de-emphasized due to the likelihood of not detecting change in these scores. Relevant subdomains, including cognitive function and lymphedema, would be expected to be captured from these four primary domains (i.e. lymphedema would be taken into account by assessing upper extremity function in a breast cancer patient). While other domains would be important to capture, including symptoms of distress and anxiety and pain, the group prioritized domains that would capture relevant subdomains and are readily modifiable by rehabilitation interventions. Creating an assessment tool that would be low burden for patients to complete and providers to administer in clinic was also a goal, limiting the scope of this project so as to improve the chances of adoption. The CRMMC therefore selected a domain-specific approach to development of the new measure.

### Determination of Trait Ranges

For this project, traits were considered to be specific self-reported functional limitations, as queried within the selected items, with the goal of application in cancer rehabilitation clinical settings. Cancer patients experience a wide range of heterogeneous impairments that affect function, and are mediated by cancer type and stage, phase of disease, treatment modalities, and underlying host conditions. Simultaneously, some impairments are common to multiple cancer types, such as fatigue, peripheral neuropathy, and radiation fibrosis.

Furthermore, major differences exist across incidence of cancer types, mortality and survival, and the extent to which each produces unique disabling effects. All the above considerations inform selection of which functions to emphasize in patient evaluation. For example, breast cancer, one of the most common malignancies, is characterized by high survivorship and high incidence of long-term effects. It is therefore crucial that the measurement tool capture specific functional limitations seen in this group. At the same time, the overall item selection must be sensitive to generalized concerns seen within the larger cancer population and, as well, sensitive to specific disabling complications seen in other types of cancer, such as neurologic tumors, sarcomas, and head and neck malignancies.

Examples of potentially meaningful traits pertinent for cancer patients for selection of items include upper limb function, fine motor dexterity, mobility, stamina, and social participation. Items also need to be relevant across age and gender, and among individuals with differing social and occupational roles, ethnic and cultural backgrounds, and socioeconomic status.

The CRMMC also recognized the need for the final selection of items to be included in the instrument to capture the full range of each function and trait, low to high, within our sample. In particular, the group wanted to avoid ceiling effects given that the majority of impaired individuals with cancer are treated in the outpatient setting and, presumably, have higher performance status than those treated in inpatient settings. This criterion would assure that the raw mix of questions would discriminate not only across high functional levels, but also for patients with lower function. Finally, the CRMMC selected individual items that would likely discriminate over a very broad arc of difficulty within its domain, thus ensuring adequate coverage of trait range and while minimizing the questionnaire’s length. Ultimately, the CRMMC agreed that each item’s capacity to discriminate over a wide performance range would be among the most heavily weighted psychometric properties used to identify what we determined to be the best performing items for inclusion in the PRO tool.

### IRT Bank Administration Mode

Three modes are available for the administration of IRT-modeled banks, CAT, generic SF, custom SF. There are pros and cons to each approach. CAT requires computerized administration and access to the CAT engine. For multi-site data collection with varying resources, this may be a substantial drawback. Ultimately, a custom SF was agreed upon given that it would be able to be adopted at sites without the resources to administer CATs and would be more clinically feasible than a longer assessment.

### Subdomain Coverage

After selecting primary domains, subdomain coverage of items was reviewed for relevant trait range and adequate discrimination over a wide range of functional performance to maximize item-level information available to clinicians. For example, in the domain of general physical function, both the ability to complete activities of daily living and aerobic exercise capacity are encompassed, albeit by very different functional tasks. Additionally, fine motor coordination is encompassed within the upper extremity domain, cognitive and activity interference is addressed in the fatigue domain, and employment issues in the domain of social participation. **Table 2** lists the domains and subdomains captured by the proposed instrument.

**Table 2:** Domains (bolded) and Sub-domains (bulleted) of the Assessment Tool.

Gross physical function	
Instrumental activities of daily living	
Aerobic/exercise capacity	
**Upper extremity function**	
Neuropathy/fine motor	
Lymphedema	
**Fatigue**	
Activity interference	
Fatigability	
Cognitive interference	
Experience	
**Social Participation**	
Family/personal	
Social/friends	
Work	

### Item Information Characteristics

Selection of specific items was the most intensive phase of the process. Each CRMMC member reviewed the more than 100 PROMIS items included in the physical function, fatigue, and social participation banks. Each member then prioritized eight items each to be considered for inclusion in the final PRO. Individual responses were combined to assess for frequently chosen items.

Each team member independently reviewed the compiled results prior to the next meeting. Several items selected by multiple investigators were included in the instrument based on consensus. If two items were similar, only one was chosen; in general, items were selected to best capture cancer-treatment related functional changes. Next, the CRMMC reviewed the individual information characteristics of each PROMIS item to identify items that performed well in the general population, their calibration slope characteristics (higher number being consistent with higher discrimination), and thresholds (reflecting their difficulty in minimizing floor and ceiling effects).

## Results

Ultimately, 21 items were selected for the SF, including six upper limb function questions, and five questions each addressing gross physical function, fatigue, and social domains. Two cross-checks were also performed prior to implementation. First, reading levels of the selected questions were analyzed, with about half of the items being written at the 3^rd^–4^th^ grade level, and the rest at the 7–12^th^ grade level. While the higher reading level of some of the questions posed a concern, upon actual review of the questions the concern was deemed relatively insignificant, especially given that the questions had already been vetted in other populations. The metabolic equivalents (METs) of the physical functions addressed by the questions were also assessed, *via* the Compendium of Physical Activities (34). While many of the test items encompassed a wide range of intensities and probable activity duration, they were found to span a range 1.8 to 9 METs, which the group felt was sufficient (**Appendix 2**).

In addition to 21 PROMIS items, the final questionnaire included eleven-point numeric ratings of pain and distress as anchors, as well as a rating of change item. The two numerical rating scales are well established. The distress scale was adapted from the National Comprehensive Cancer Center’s Distress Thermometer, which evaluates the supportive care services cancer patients may need (35). The pain rating scale is ubiquitous and used both clinically and in research. The perceived change rating was to be assessed by asking returning patients whether they felt better, the same, or worse than they had at their previous visit.

Clinicians were also required to provide additional demographic data about the patients on the day that the patient was seen and completed the questionnaire. These data included age, gender, oncologic diagnosis, disease stage, presence of bone or central nervous system metastases, treatments rendered, presence of polyneuropathy, non-oncologic comorbidities that might impact function (musculoskeletal, neurologic, cardiopulmonary, psychiatric), and body mass index (**Appendix 3**).

**Figure 1:**
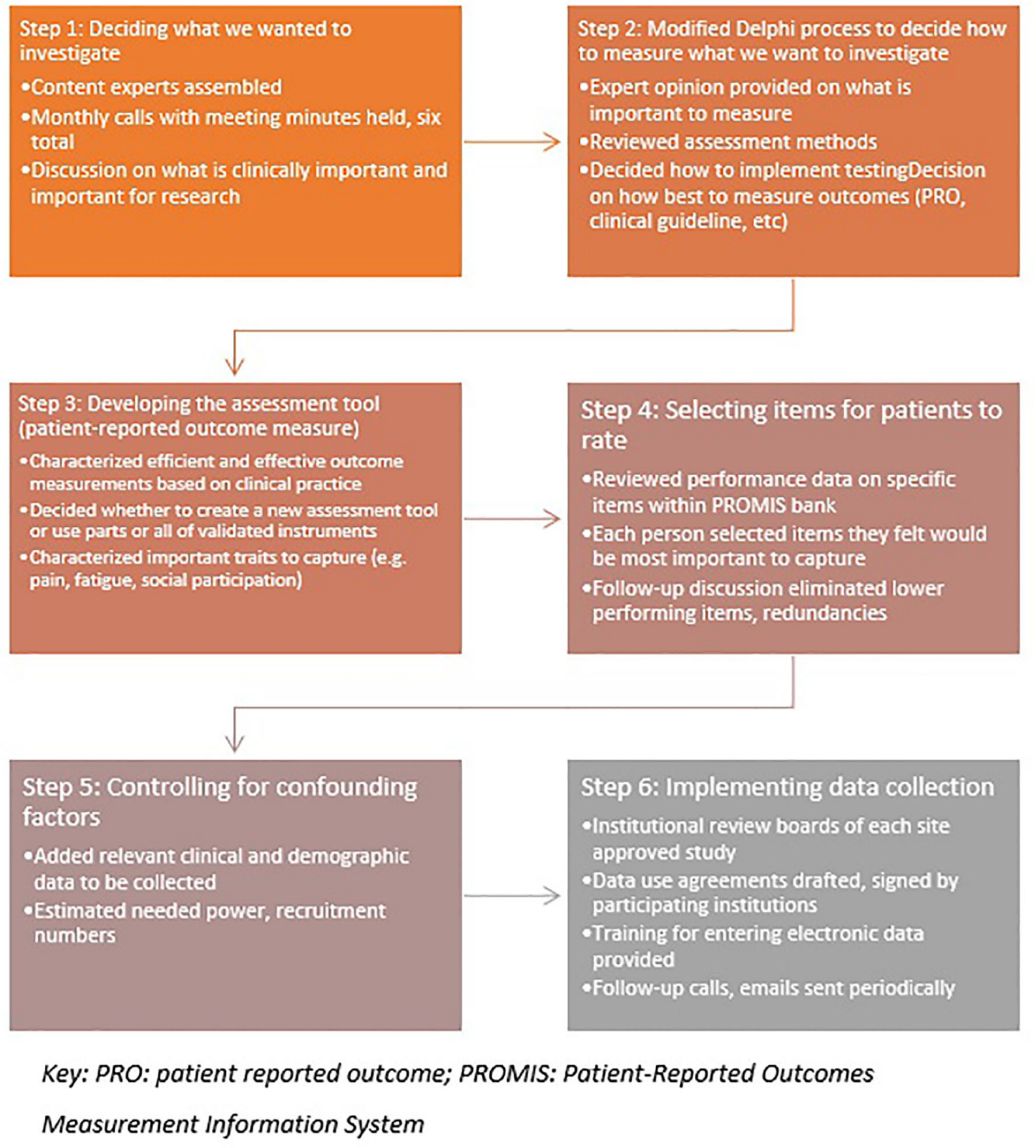
Stepwise Modified Delphi Process (Uploaded Separately).

Finally, the group chose to record performance status, a gross measure of physical independence used by oncologists to determine if a patient is physically strong enough to receive further treatment. While these measures do not sufficiently reflect a patient’s overall status or change in function, it was felt that including KPS and ECOG ratings could be useful for research purposes and anchoring would be welcomed by oncologists. A hypothesized area of research is that this new measurement tool may provide a better reflection of a patient’s ability to tolerate cancer treatment than current measures. A workflow of the steps taken in this modified Delphi process is listed in **Figure 1**.

## Discussion

The CRMMC developed the SF with the goal of accurately reflecting cancer patient status across the continuum of disease, regardless of cancer type, treatment, or location. The group decided on candidate items that reflected domains and subdomains encompassing a broad range of cancer-related function, including gross and fine motor activity, fatigue, cognition, social participation, and activities of daily living. This process is unique in its evidence-based, structured, and expert-derived nature. **Table 3** outlines the processes described previously and associated challenges.

**Table 3:** Process of Developing the Assessment Tool.

Task	Methods used	Limitations/Suggested alternatives
Delineation of measurement priorities and specifics	Discussion to establish consensus	May be difficult to achieve goal of feasible in clinical/research setting and effective in measuring function
Selection of measurement approach	Literature review Enumerate options Discussion to establish consensus	Resource constraints limit options, and assessment tool must be translatable to different practice models.
Selection of domains	Literature review Stakeholder assessments Enumerate domain options Discussion to narrow options Modified Delphi process	Limited engagement of allied health providers, may be difficult to hone in on only a few domains
Determination of relevant domain trait ranges	Literature review Discussion to narrow candidate domains Modified Delphi process	Clinical practice is biased towards lower-functioning patients, making it difficult to find relevant items to evaluate higher levels of function
Selection of instruments	Literature review. Identification of instruments in current use (national, institutional, across disciplines) and their limitations Discussion to determine general *vs.* domain, generic *vs.* condition specific, IRT *vs.* legacy Determination of feasible assessment modes Enumeration of candidate instruments Modified Delphi process	May need multiple assessment modes
Selection of items	Group discussion to prioritize subdomains Review of item information characteristics and trait discrimination range Identify candidate items Modified Delphi process Narrow candidate item pool Group discussion Repeat modified Delphi process	Difficult to discern value amongst similar items, need to correlate items with other factors (METs, ICF, etc.)
Vetting of PRO performance	Prospective data collection, PRO, and clinical/demographic anchors IRT estimation of item information Modeling to estimate trait estimates with clinical anchors Modified Delphi process, as needed, for item culling	Requires institutional review board approval, resources/funding, data management expertise, power analysis should be performed before data collection

IRT, item response theory; METs, metabolic equivalents; ICF, the International Classification of Functioning; PRO, patient-reported outcomes.

Currently, a multi-center study is underway in outpatient cancer rehabilitation clinics at the CRMMC institutions in which patients answer the instrument’s items and scores are calculated. Each patient is assigned a study identification number to capture changes over time. The study has been approved by the Institutional Research Boards of all of the participating institutions, and patients are required to provide consent to have their data collected if required by the institution. The University of Michigan is the coordinating site and is responsible for securely storing the data, development of a tablet-based form of the assessment tool, and coordinating data use agreements.

This study requires the participation of multiple CMMRC sites for two reasons. The first is that a large cohort (>500) of patients is needed to permit an adequate statistical analysis of the numerous covariates that must be considered. The second is that practice patterns vary by between the sites with one perhaps seeing more of a specific tumor diagnosis or managing different symptoms than the others. This will also help to capture at-risk populations, including racial and ethnic minorities and rural cancer patients who travel to large cancer centers for their care.

Any adult patient seen in an outpatient cancer rehabilitation medicine clinic will be eligible to participate. Patients may range from those being in the midst of active treatment, to long-term survivors, to individuals with advanced disease. Excluded subjects include those cognitive or communication (including language barrier) challenges interfering with their ability to complete the PRO tool.

Psychometric vetting of the instrument will take many forms, not all of which have been fleshed out during the data-collection period. First, the tool will be evaluated grossly to determine if the items exhibit variation between patients depending on their condition, and to evaluate longitudinal construct validity by assessing if the instrument captures change as patients are seen over time. Additionally, item performance will be evaluated with the goal of culling any items that are non-discriminative or offer no incremental trait or subdomain information. Distribution and anchor-based methods will be applied to characterize responsiveness and regression analyses performed to evaluate items *vis-à-vis* patient characteristics such as the presence of active and/or metastatic cancer.

Other planned methods of evaluation include comparing the performance of this instrument to existing legacy tools. Paper *versus* tablet-based responses will also be analyzed to control for mode effects and ensure that they can be administered in different ways.

## Future Directions, Relevance, and Limitations

This project is novel because it seeks to address a persistent gap in cancer patient care by developing an assessment tool that evaluates multiple domains across a wide range of trait characteristics. The process to develop this assessment tool provides a framework for other providers to follow.

It is essential that cancer rehabilitation research becomes more rigorous and involves large subject numbers if it is to effectively study this population. Oncology research often involves multi-site Phase 2 and Phase 3 trials, and cancer rehabilitation research must be held to this standard if it is to be widely accepted in oncology care. The CRMMC project advances cancer rehabilitation research in this direction by compiling a large multi-institution data set and lays the groundwork for future, collaborative clinical trials. Dissemination of the final instrument will take place in multiple ways, including presentations at academic conferences and specific sites, engaging key stakeholders including oncology and clinical trial groups, and open access publishing of results to make the assessment tool and supporting data readily accessible.

While limitations of this instrument can be better assessed after the data collection phase, some are already known. First, important aspects of function may have been omitted as a result of limiting the number of items to improve clinical feasibility and minimize patient burden. For example, we had to make decisions about which PRO domains to focus upon and, prioritized physical function, fatigue, and social participation. Other important domains, such as cognitive function, are only addressed as subdomains. Involving multiple cancer rehabilitation physiatrists from various practice settings may minimize this risk.

Additionally, because this project was conceived in recognition of the crucial within-specialty need for a reliable and valid measurement construct for tracking the function of cancer patients engaged in cancer rehabilitation care, the development of the tool has relied on physiatry expertise. Involvement of other stakeholders, such as patients and non-physiatry clinicians, was beyond the present scope.

Other limitations include that while the CRMMC had the advantage of access to existing item performance data for the general population, it is possible that some items may perform differently in patients with cancer. Another is that although the CRMMC made great efforts to have a common approach to the completion of the clinical data collection forms, it is still possible that inter-investigator/institution differences can occur. It remains to be seen if this tool would perform differently in a large urban academic center compared to a community or rural setting. Finally, this study focuses only on patient-reported outcomes for practical reasons outlined extensively earlier. Additional approaches may provide potentially useful information.

## Data Availability Statement

The original contributions presented in the study are included in the article/**Supplementary Material**. Further inquiries can be directed to the corresponding author.

## Ethics Statement

The study has been approved by the Institutional Research Boards of all of the participating institutions, and patients are required to provide consent to have their data collected if required by the institution.

## Author Contributions

All authors were significantly involved in the outlining and writing of this manuscript. Authors SRS, MV, DZ, AC, EW, CC, LG, MH, and SS were involved in the process of selecting items for an assessment tool described in the manuscript. Author GJ was involved in creating a usable framework for testing the assessment tool. All authors contributed to the article and approved the submitted version.

## Funding

Portions of this Study were funded with the support of a grant from the Foundation for PM&R.

## Conflict of Interest

The authors declare that the research was conducted in the absence of any commercial or financial relationships that could be construed as a potential conflict of interest.
