# Supplementary material for: The Cancer Rehabilitation Medicine Metrics Consortium: A Path to Enhanced, Multi-Site Outcome Assessment to Enhance Care and Demonstrate Value
Source: Front Oncol. 2021 Feb 3;10:625700. doi: 10.3389/fonc.2020.625700 (PMC7887295; doi:10.3389/fonc.2020.625700)
Supplement: Supplementary file 1 [file DataSheet_1.docx]

**Appendix 1: Meeting dates and topics**

| Date  11/20/2015  11/30/2015  3/4/2016  4/18/2016  6/8/2016  7/22/2016  8/9/2016  12/19/2016  1/13/2017  2/17/2017 | Topics Discussed  Measurement priorities, measurement approach (PRO), and general conceptual framework. Decided on PROMIS as a basis for the PRO and what domains to address  Discussed feasibility, how to select items for inclusion. Assigned task of reviewing PROMIS items to be voted on for potential inclusion  Reviewed voting results, and desire to capture a wide trait range and multiple subdomains  New member (UPMC) added. Discussed framework for data sharing  Discussed potentially competing projects from other groups. Reviewed what clinical correlates we want to record  Discussed reading level required for each item, and sample size required for the project  Reviewed MET values of each item, discussed logistics of seeking ethical board approval at each site, and began refining the clinical intake form. Considered alternate language forms.  Discussed data use agreements and status of ethical board approval. Discussed mode of patients completing questionnaires.  Reviewed ethical board and data use agreement progress. Further refined clinical intake form.  Finalized clinical intake form. Reviewed ethical review board progress and each site’s mode of questionnaire in anticipation of enrolling patients soon. |
| --- | --- |

*Key: PRO-patient reported outcome; PROMIS-Patient-Reported Outcome Measurement Information System; UPMC-University of Pittsburgh Medical Center; MET-metabolic equivalent*

**Appendix 2**: **Metabolic equivalents per each item in the questionnaire**

| **Metabolic Equivalents**  **(estimated)** | **Physical Function--Gross Motor** |
| --- | --- |
| **2.5-4.5** | Are you able to go for a walk of at least 15 minutes? |
| **8-climbing**  **3.5- descending** | Are you able to go up and down stairs at a normal pace? |
| **6+** | Are you able to run a short distance, such as to catch a bus? |
| **1.8** | Are you able to sit on and get up from the toilet? |
| **N/A-duration question** | Does your health now limit you in doing two hours of physical labor? |
|  | **Physical Function--Upper Limb** |
| **2.5** | Are you able to dress yourself, including tying shoelaces and buttoning your clothes? |
| **4.0-5.0**  **(extrapolated from similar items)** | Are you able to carry a laundry basket up a flight of stairs? |
| **3.3** | Are you able to make a bed, including spreading and tucking in bed sheets? |
| **2.0-3.3 (extrapolated from similar items)** | Are you able to reach into a high cupboard? |
| **2.0** | Are you able to shave your face or apply make-up? |
| **3.5 - 9** | Does your health now limit you in doing heavy work around the house like scrubbing floors, or lifting or moving heavy furniture? |

**Appendix 3: The 21 PROMIS items administered to patients and clinical intake form**

| Domain | Item | Response Categories |
| --- | --- | --- |
| Physical Function | Are you able to go for a walk of at least 15 minutes? (PF1) | Without any difficulty (5), With a little difficulty (4), With some difficulty (3), With much difficulty (2), Unable to do (1) |
|  | Are you able to go up and down stairs at a normal pace? (PF2) |  |
|  | Are you able to run a short distance, such as to catch a bus? (PF3) |  |
|  | Are you able to sit on and get up from the toilet? (PF4) |  |
|  | Are you able to dress yourself, including tying shoelaces and buttoning your clothes? (PF5) |  |
|  | Are you able to carry a laundry basket up a flight of stairs? (PF6) |  |
|  | Are you able to make a bed, including spreading and tucking in bed sheets? (PF7) |  |
|  | Are you able to reach into a high cupboard? (PF8) |  |
|  | Are you able to shave your face or apply make-up (PF9) |  |
|  | Does your health now limit you in doing two hours of physical labor? (PF10) | Not at all (5), A little bit (4), Somewhat (3), Quite a lot (2), Cannot do (1) |
|  | Does your health now limit you in doing heavy work around the house like scrubbing floors, or lifting or moving heavy furniture? (PF11) |  |
| Fatigue | In the past 7 days, how often were you too tired to think clearly? (F1) | Never (5), Rarely (4), Sometimes (3), Often (2), Always (1) |
|  | In the past 7 days, how often did you have trouble finishing things because of your fatigue? (F2) |  |
|  | In the past 7 days, how often did you have to limit your social activities because of your fatigue? (F3) |  |
|  | In the past 7 days, how often were you bothered by your fatigue? (F4) |  |
|  | In the past 7 days, how often did your fatigue make it difficult to plan activities ahead of time? (F5) |  |
| Social Participation | I have trouble doing all of my regular leisure activities with others. (SR1) | Never (5), Rarely (4), Sometimes (3), Often (2), Always (1) |
|  | I have to do my work for shorter periods of time than usual. (SR2) |  |
|  | I have trouble doing all of the family activities that I want to do. (SR3) |  |
|  | I have trouble doing all of the work that is really important to me (including work at home). (SR4) |  |
|  | I have trouble doing all of the activities with friends that I want to do. (SR5) |  |

Portion filled out by the physician:

Visit type:

🞎 New

🞎 Return Visit

Cancer management stage:

🞎 Primary Treatment

🞎 Recurrence

🞎 No evidence of disease

Age _________

Gender: Male Female Other

Type of cancer (check all that apply):

🞎 Breast

🞎 Head/Neck, non-thyroid

🞎 Thyroid

🞎 Prostate

🞎 Brain

🞎 Sarcoma

🞎 Melanoma

🞎 Colorectal

🞎 Bladder/Ureteral

🞎 Renal

🞎 Lung

🞎 Gynecologic

🞎 Multiple myeloma

🞎 Leukemia, no alloBMT

🞎 Lymphoma, no alloBMT

🞎 History of allogeneic BMT

🞎 Other

Active disease? 🞎Yes 🞎No

Presence of non-regional metastases? 🞎Yes 🞎No

Location of metastases?

🞎 Brain

🞎 Spinal cord

🞎 Leptomeningeal

🞎 Bone

Oncologic treatment history:

Chemotherapy: 🞎Active 🞎Previous 🞎Never

Hormone-associated therapy: 🞎Active 🞎Previous 🞎Never

Immunotherapy: 🞎Active 🞎Previous 🞎Never

Radiation therapy: 🞎Active 🞎Previous 🞎Never

Surgery 🞎Within 4 weeks 🞎4+ weeks ago🞎Never

Other sources of impairment:

Clinical symptoms of peripheral neuropathy? 🞎Yes 🞎No

Major non-cancer sources of impairment?

Musculoskeletal 🞎None/minimal 🞎Moderate 🞎Severe

Neurologic 🞎None/minimal 🞎Moderate 🞎Severe

Cardiopulmonary 🞎None/minimal 🞎Moderate 🞎Severe

Psychiatric 🞎None/minimal 🞎Moderate 🞎Severe

BMI:

🞎 Less than 18.5

🞎 18.5-25

🞎 25-30

🞎 30-35

🞎 35-40

🞎 40-45

Ambulation status: 🞎Independent 🞎Mod-I 🞎Wheelchair

Performance Status:

Karnofsky:

🞎100 🞎90 🞎80 🞎70 🞎60 🞎50 🞎40 🞎30 🞎20 🞎10 🞎0

ECOG:

🞎0 🞎1 🞎2 🞎3 🞎4 🞎5

*Key: PROMIS=Patient-Reported Outcome Measurement Information System; PF=physical function; F=fatigue; SR=ability to participate in social roles and activities; alloBMT=allogeneic bone marrow transplantation; BMI=body mass index; Mod-I=modified independent; Karnofsky=Karnofsky Performance Status Scale; ECOG=Eastern Cooperative Oncology Group Performance Status scale*
